# Fracture Analysis of High-Strength Screw for Highway Construction

**DOI:** 10.3390/ma14071599

**Published:** 2021-03-25

**Authors:** Andrej Dubec, Petra Kováčiková, Jan Krmela, Vladimíra Krmelová, Artem Artyukhov

**Affiliations:** 1 Department of Material Engineering, Faculty of Industrial Technologies in Púchov, Alexander Dubček University of Trenčín, Ivana Krasku 491/30, 020 01 Púchov, Slovakia; andrej.dubec@tnuni.sk; 2 Department of Numerical Methods and Computational Modeling, Faculty of Industrial Technologies in Púchov, Alexander Dubček University of Trenčín, Ivana Krasku 491/30, 020 01 Púchov, Slovakia; petra.kovacikova@tnuni.sk; 3 Department of Material Technologies and Environment, Faculty of Industrial Technologies in Púchov, Alexander Dubček University of Trenčín, Ivana Krasku 491/30, 020 01 Púchov, Slovakia; vladimira.krmelova@tnuni.sk; 4 Department of Marketing, Oleg Balatskyi Academic and Research Institute of Finance, Economics and Management, Sumy State University, 2 Rymskogo-Korsakova St., 40007 Sumy, Ukraine; a.artyukhov@pohnp.sumdu.edu.ua

**Keywords:** fracture analysis, scanning electron microscopy, high-strength screw, intergranular fracture, tempering embrittlement

## Abstract

High-strength screws represent one of the main joining or fastening components which are commonly used in the process of installation of frame constructions for information boards or signposts, relating to the traffic roads. The control of the production process may not always be a sufficient method for ensuring road safety. The backward investigation and control of the screw material processing seems to be the one of the most important procedures when there is the occurrence of any failure during the operation of the screw. This paper is mainly focused on the analysis of the failure of the high-strength screw of 10.9 grade with M diameter of 27 × 3 and a shank length of 64 mm. The mentioned and investigated screw was used as a fastener in a highway frame construction. In the paper, there is mainly the analysis of the material for a broken screw in terms of the material micropurity, the material microstructure, the surface treatment as well as chemical composition. The evaluation was based on investigation by optical microscopy, scanning electron microscopy and energy dispersive spectroscopy. Important knowledge and results were also obtained due to information on micromorphology and material contrast of the fracture surface resulting from fractographic analysis, using the method of scanning electron microscopy. In the case of the production of the high-strength screws, the tempering stands for the decisive or crucial process of heat treatment because the given process can ensure a decrease in hardness, while the required ductile properties of the material are kept and this is also reflected in the increase of strength and micromorphology of the fracture surface. From the aspect of micropurity, inclusions of critical size or distribution were not identified in the material, referring to Czech standard ČSN ISO 4967 (420471). The microstructure corresponds to tempered martensite, but the fracture surface of the broken screw was based on an intercrystalline micromechanism, which is undesirable for the given type of component. Combined with the measurement of the HV1 (Vickers hardness at a load of 1 kg) from the edge to the central area of the screw, the analysis revealed the significant drawbacks in the heat treatment of the high-strength screw.

## 1. Introduction

Highway steel frame constructions have the function of a supporting structural part for information and signaling boards or signposts in relation to the traffic roads. Particular attention is paid to the keeping of all relevant or appropriate requirements, from installation to maintenance [1,2]. In relation to the performance of high-strength screws, excessive attention has to be paid to their functionality as well as reliability and safety. The parameters of steel constructions have to be suitable from the aspect of the specific snow and wind conditions to which the given screws are exposed. The constructions are mounted with various screw or welded joints and they have to represent the required quality and accuracy [2]. During the operation of various constructions and equipment under variable oscillating loading, the occurrence of fractures of construction structural elements and components is quite common even under the stresses that are considerably lower than the yield point of the material and therefore, the production process must be innovated, improved and optimized constantly [1,2].

Materials for production of screws undergo various production technologies to improve their properties but the inadequate selection of material as well as inappropriate manufacturing procedure can lead to the damage or rupture as well as subsequent fracture or failure of the material [3]. This type of introduced structural parts or components is mainly exposed to a particular cyclic loading and this mentioned fact has to be taken into account in relation to the selection and testing procedure of the material [3,4]. Highway information boards or signposts often have a greater weight as well as geometric dimensions. In most cases, a combination of several screws is used to join various information boards or signposts. Considering the fact mentioned hereinbefore, it is important to pay attention to the geometric and material aspects of screw processing in order to avoid the rupture and failure, relating to the whole cross-section of screw because the given phenomenon could lead to the shear stress effect on the lower number of operating cross-sections of the remaining screws [5,6,7,8]. In this mentioned case, an unstable screw joining could occur and the information board could fall on the road during traffic and it could have fatal consequences [1,2,3,4,5,6,7,8].

High-strength steels are particularly susceptible to brittleness of the material, while the given embrittlement is seriously dangerous. Depending on the chemical composition of the steel and the tempering temperatures or the course of the thermal process, there can be the occurrence of the changes in the microstructure with a negative effect if the material reaches the region of the tempering embrittlement [1,2]. Based on the way of the embrittlement occurrence, the transcrystalline or intercrystalline rupture mechanism can be observed in relation to the fracture surface. In comparison with the transcrystalline fission rupture, the fractures created by intercrystalline ductile rupture belong to the group of low-energy fractures where the less energy is required [9,10,11,12]. The formation of such fractures means that the conditions for the ductile rupture were kept only for narrow zones which are adjacent to the grain boundaries. The embrittlement can be caused by inappropriate heat treatment as well as by infringement of the technological procedure for surface treatment [13].

Proper tempering temperature has a significant effect on the tempering embrittlement. The given tempering temperature represents a decisive or crucial factor in the extent of structural changes and changes related to the mechanical properties of stainless steels. The values of notch and fracture toughness are decreased in the steel with the occurrence of tempering embrittlement and, moreover, there is a higher susceptibility to corrosion cracking or hydrogen embrittlement [14,15,16]. Nickel chromium and manganese chromium steels in particular tend to be brittle because of the toughness decrease even during the slow cooling from the tempering temperature in comparison with manganese, nickel and chromium steels, which tend to be brittle only when they are held at a given temperature for a long time. Carbides or other compounds, which are dissolved in the ferrite at a higher temperature, are segregated by cooling and it leads to the tempering embrittlement [14,15,16,17,18]. There is no occurrence of the tempering embrittlement during rapid cooling. The described embrittlement can be eliminated with molybdenum and partly with tungsten and vanadium. Therefore, the addition of Mo is particularly important in steels which are used for the production of large cross-section components or for components that are permanently exposed to the critical temperatures. At low temperatures, commonly between 350 and 450 °C, low-temperature tempering embrittlement occurs [14,15,16,17,18].

During slow cooling from the tempering temperature, embrittlement can occur due to the development of the so-called anisothermal constituent part. The susceptibility of steels to tempering embrittlement is based on the chemical composition of steels, especially on the content of the surface-active elements, such as P, S, Sn, As, Sb and others [19,20]. Low tempering embrittlement is accompanied by transcrystalline fission rupture on the fracture surfaces and the reason is hidden in the inappropriate size, shape and distribution of the cementite particles. In the case of intercrystalline decohesion along the prior austenitic grains, the occurrence of low-temperature tempering brittleness is closely connected with the segregation of the surface-active elements towards the boundaries during the austenitizing heating and moreover, it is accompanied with the simultaneous precipitation of carbides in these areas [19,20,21].

High-temperature tempering embrittlement and the maximum decrease in toughness occur at a relatively high tempering temperature, which is around 500 °C. The high-temperature tempering can be carried out under the isothermal or anisothermal conditions of the heat treatment [22,23]. The main reason for occurrence of the high-temperature embrittlement is associated with the segregation of the surface-active elements at the boundaries of the prior austenitic grains, especially during tempering or slow cooling from the tempering temperature. In the most cases, the segregation of alloying elements, such as Ni, Mn, Cr and Si, is considered as a factor which leads to the reduction in the cohesive strength of grain boundaries. The degradation effects are more noticeable and significant in relation to coarse-grained steels, which have a high yield point [22,23,24].

The main objective of this paper is to obtain knowledge from individual applied analyses and to summarize the results in order to gain the information on the reasons of failure of a high-strength screw which was mounted in a highway frame construction. The resulting information can help to identify the individual procedures in manufacturing process because inappropriate manufacturing procedures can lead to defects in the microstructure.

## 2. Material and Experimental Methods

Relating to the mechanical joints of steel constructions, the fasteners, including screws, have to fulfill the requirements which are prescribed in the standards. The material of the screws and screw joints has to meet the requirements of STN EN 1993-1-1 and STN EN 1993-1-8 and STN EN 1090-2 + A1. Non-prestressed screw joints, which are made of carbon steels, alloyed steels and austenitic stainless steels have to meet the requirements according to STN EN 15048-1. High-strength prestressed screw joints have to meet the requirements according to STN EN 14399-1 [1]. The M 27 screw of 10.9 strength grade has a commonly used geometry, a full threaded shank and a hexagonal head. It is made of high-strength 41Cr4 (1.7035) chrome steel with a chemical composition shown in Table 1 and it is mainly used as an exterior fastener for steel constructions. The basic mechanical properties of 41Cr4 steel are given in Table 2. For heat-treated high-strength screws, the continuous control of the hardness is very important and for 10.9 strength grade, the surface hardness is not allowed to be higher than 390 HV [2]. In practice, 34Cr4 (1.7033) and 37Cr4 (1.7034) steels can also be used for the production of joining components. Two types of anticorrosion surface protection are the most often used, namely galvanic plating or hot-dip zinc plating, depending on the thickness of the protective layer and the type of application as well as on the aggressiveness of the surrounding environment [25].

**Figure 1:**
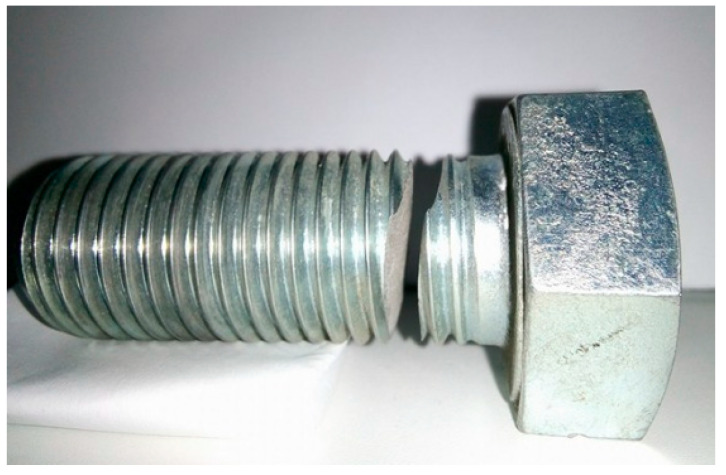
Operational fracture of high-strength screw.

The investigated broken M 27 × 3 screw with a shank length of 64 mm (Figure 1) was used to fasten a highway construction in combination with a certain number of screws of the same designation but the exact number of joining screws is not known. When mounted in an accurate way, the loading of screw was based on shear stress in one area of the screw shank. If the prescribed 1524 Nm tightening torque, at which the magnitude of the axial force in the screw is 291,534 N, is not met, an additional value of the tensile force can be induced in the screw material. The stressed cross-section of screw was located in the threaded part of the screw because this type of screw is structurally designed in such a way that the thread is made just below the screw head. The important information obtained was based on the fact that only one of all the screws, which were mounted in the highway construction, was broken. To solve the cause of the failure of the connecting element of the highway structure, only two fractures from the given screw were delivered, which represented a piece and a counter piece. One piece of identical undamaged screw, which was not yet in operation, was also delivered. The whole process of research was closely bound only to this volume of material. For this reason, it has not been possible to carry out extensive research into the mechanical properties of the material from which the screws were made. Therefore, microscopic methods were chosen for the realization of the experimental part, as their advantage is the work with a small volume of material. The method of fracture surfaces comparison was chosen to give an information of the correctness of the heat treatment of the screw. Specifically, the fracture areas of the screw operational quarry, the laboratory quarry of the undamaged identic screw and the laboratory quarry of the broken screw from the area outside the operational quarry were investigated. To obtain these fracture surfaces, samples were made from the damaged and undamaged screw, which could be torn on a tearing machine by applying tensile force. However, the volume of material did not allow the production of samples for the tensile test according to the standard, therefore it was not possible to have data on the mechanical properties of the screw obtained by the tensile test. By comparing the resulting fracture surfaces, it was possible to determine the suitability of the microstructure after heat treatment, which is specified by the standard. Analysis of the screw material by optical microscopy was chosen to evaluate the effect of inclusions in the steel on the occurrence of screw failure. Samples has been removed in the longitudinal direction from the area close to the fracture. These samples were prepared by successive sanding of the material using sandpapers. After completion of the metallographic sanding, the samples were polished in ethanol medium. The polishing substrate with the addition of an abrasive with a grain size of 2 µm was used, for the first phase of polishing. The polishing substrate with the addition of an abrasive with a grain size of 0.7 µm was used, for the final phase of polishing. Samples prepared by this procedure were used in a polished state to evaluate inclusions according to the standard. In the next step, the samples were etched by etchant Nital 2% in order to be able to evaluate the microstructure after heat treatment and also to perform a Vickers hardness measurement. Sample preparation for SEM analysis was focused on ultrasonic cleaning of fracture surfaces in isopropyl alcohol. Due to the fact that the samples were conductive, no additional conductive treatment of the analyzed areas was necessary. The fracture areas were analyzed in the secondary electrons (SE) mode to evaluate the micromorphology and the material contrast in the back scattered electrons (BSE) mode was also evaluated. SE and BSE detectors were used simultaneously in dual display to obtain complex information from the fracture surface. The samples were evaluated under high vacuum. The energy dispersive spectroscopy (EDS) method was used to obtain information on the chemical composition in microareas. Specifically, the chemical composition analysis of the selected area was used. The result of this analysis is a qualitative and quantitative evaluation of chemical elements in the given material. This type of analysis also provides information on the approximate location of the chemical element with respect to the shape of the selected area.

**Figure 2:**
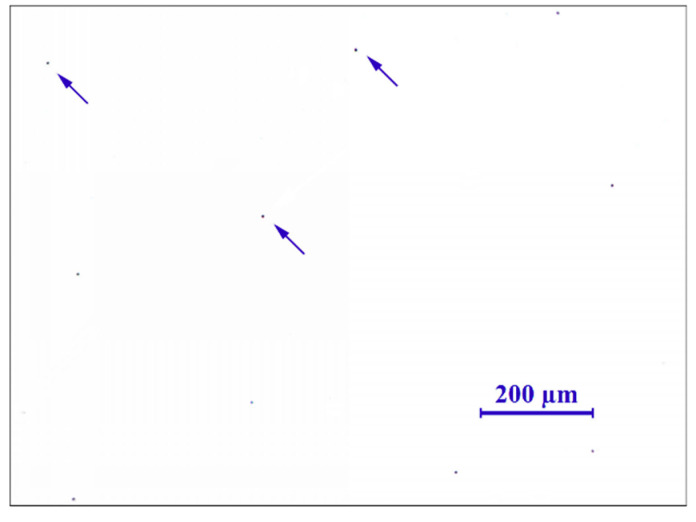
Local occurrence of the oxide inclusion.

**Figure 3:**
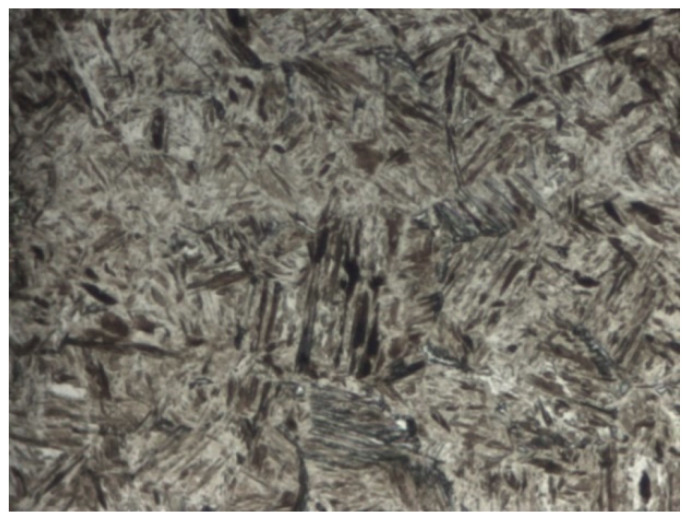
Microstructure of the tempered martensite.

**Figure 4:**
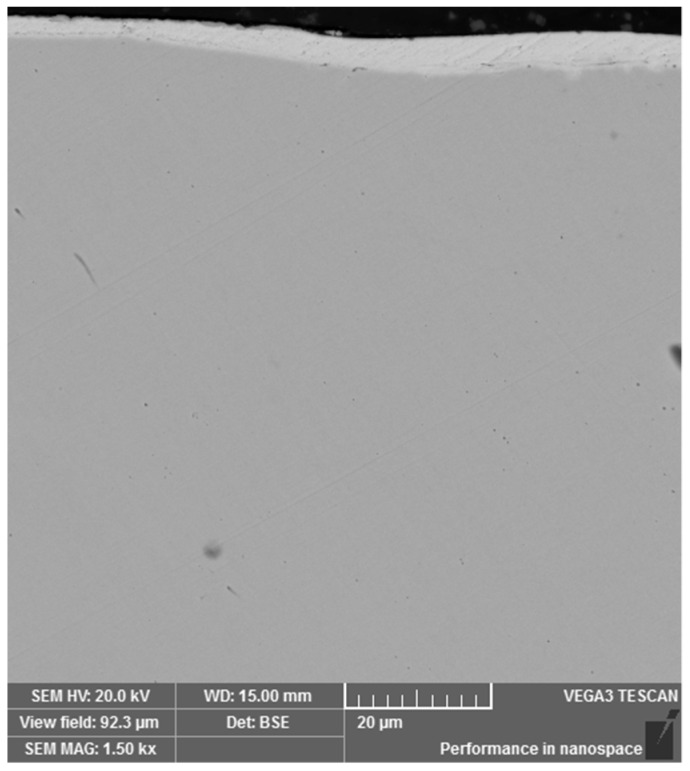
Protective Zn surface layer.

To be able to evaluate the micropurity, the microstructure and the surface treatment of the broken screw, a sample from the longitudinal section was taken from the area close to the fracture surface. The gaining of the given information was connected with the preparation of the samples in a metallographic way. Analysis of micropurity, which was performed by optical microscopy, showed that high-purity steel with local occurrence of subcritical oxides was used for the production of the high-strength screw of 10.9 strength grade. Oxide inclusions (indicated with arrows in Figure 2) were observed at 100× magnification and their size was evaluated as well as assigned to the grade of 1, referring to the standard series based on standard ČSN ISO 4967 (420471). At the mentioned magnification, which is prescribed by the mentioned standard, there was not any occurrence of other types of inclusions [27]. After etching of the polished surface with 2% Nital etchant, the microstructure of the screw was observed and it corresponded to the tempered martensite (Figure 3). This type of microstructure can be achieved in steel by quenching and subsequent tempering and it represents the suitable selection of the heat treatment technology for this type of joining component. In the further part of the experiment, it was necessary to perform a hardness measurement analysis and microstructure morphology analysis by scanning electron microscopy in order to determine the exact type of bainitic structure, which mainly depends on the tempering temperature used. In the central area of the screw, the microstructure was mainly based on the direction of plastic deforming of the semiproduct, which was used for subsequent production of final screw component. On the other side, there was not any significant change of the direction observed in the marginal part. In the polished state, the presence of a surface layer was observed on the surface of the investigated sample. This layer was not uniform and its thickness ranged from 2.7 to 5.5 µm (Figure 4). Including overall mapping analysis, energy dispersive spectroscopy was applied for investigation of the sample and it is important to point out that there was the Zn layer recognized. The defect sites of the surface layer were observed at several areas. In the threaded part, the layer was discontinuous with its complete absence in the individual microlocalities.

**Figure 5:**
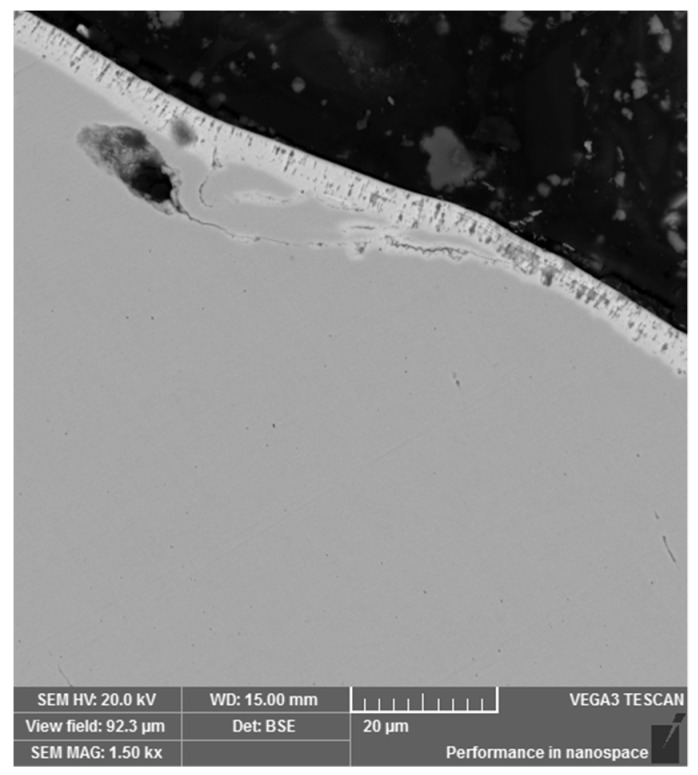
Lap (fold)—area with defect of Zn surface layer.

**Figure 6:**
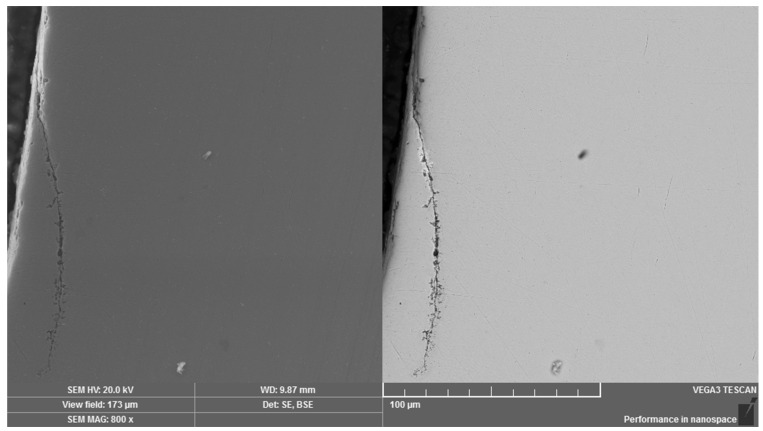
Change in direction of microcrack propagation towards the surface of thread part. Dual representation of the analyzed area: (**left**) SE mode, (**right)** BSE mode.

In addition, microcrack propagation through the overall thickness as well as peeling of the surface layer in several areas were determined. The occurrence of folds was observed on the surface of the rolled material and the given areas with laps can be considered as the consequence of the threads production by rolling technology (Figure 5). Moreover, microcavities could also be observed in these mentioned areas. In relation to the areas with the absence of the Zn layer, there were the visible sites with microcrack initiation and the given microcracks were propagated into the core of the material at an angle value which was close to 45°. If such an angle of the surface microcrack propagation remains, it can significantly increase the risk of weakening in relation to the load-bearing cross-section of the high-strength screw. When the microcrack changes the direction of its propagation towards the surface of the material (Figure 6), the surface of material is peeled off during further microcrack propagation. This process can significantly increase the surface roughness of the thread contact surfaces. In addition, the released material moves freely when the screw is tightened between the threaded areas of the screw and the nut. It is also necessary to point out that there is the change in the surface roughness of the contact surfaces. The surface microcracks were recognized in the area of the thread crest as well as in the area of the thread root. The microspace, which occurred by the propagation of microcracks in the material, was filled with corrosive elements and Zn.

## 3. Results

**Figure 7:**
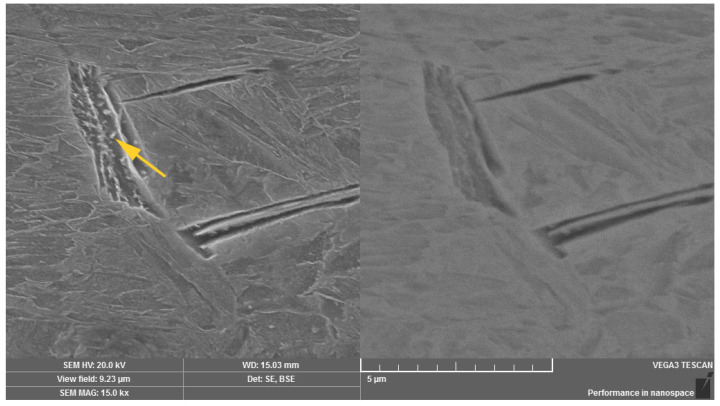
Occurrence of ε-carbide in the plate of the bainitic ferrite. Dual representation of the analyzed area: (**left**) SE mode, (**right)** BSE mode.

**Figure 8:**
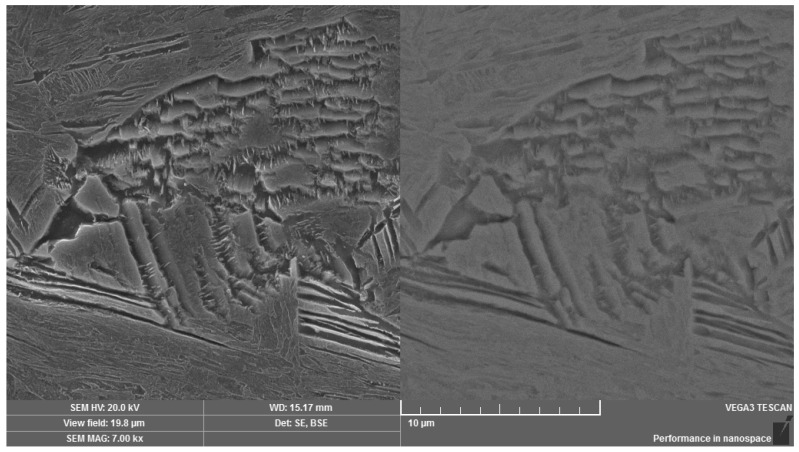
Occurrence of carbide formations in the structure of lower bainite. Dual representation of the analyzed area: (**left**) SE mode, (**right)** BSE mode.

**Figure 9:**
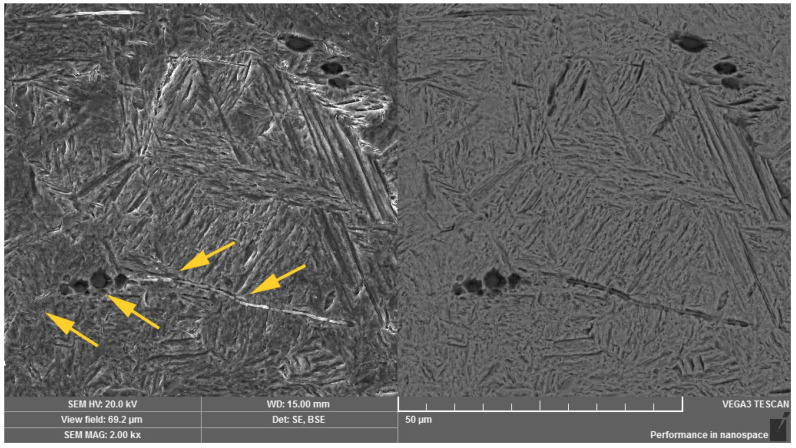
Oxide inclusions on the basis of Al, Si and MnS. Dual representation of the analyzed area: (**left**) SE mode, (**right)** BSE mode.

**Figure 10:**
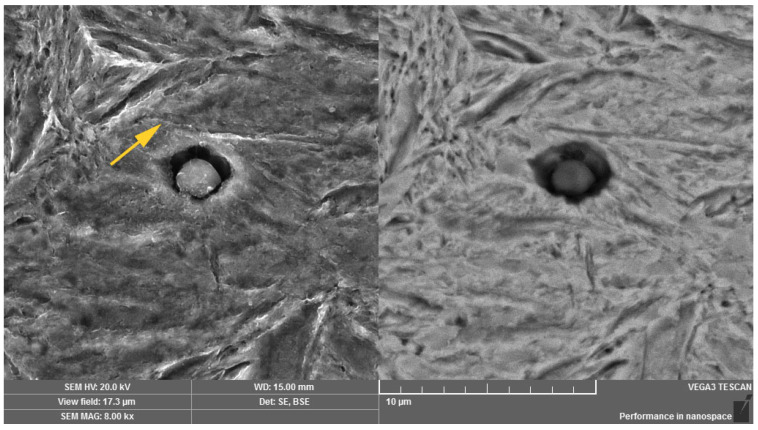
Complex sulfoxides close to the grain boundaries. Dual representation of the analyzed area: (**left**) SE mode, (**right)** BSE mode.

Using the method of scanning electron microscopy, the micromorphology of the tempered microstructure in the mode of secondary electrons and the material contrast of the analyzed areas in the mode of backscattered electrons were observed in more detailed way. The method of sequential etching, using the 2% Nital etchant, was selected for an accurate visualization and subsequent identification of the bainitic structure. By this mentioned procedure, it was possible to observe the presence of individual bainitic ferrite plates in which ε-carbide formations (indicated with arrows in Figure 7) were distributed. The more intense effect of the etchant led to more significant etching of the ferritic phase of the bainitic structure as well as more visible image of the ε-carbide formations. These microareas are visible in Figure 8. Thicker plates of bainitic ferrite can be observed quite close to the grain boundaries. The given microstructure characteristics correspond to the occurrence of lower bainite, i.e., martensitic structure tempered at a lower temperature. In the etched microstructure, the occurrence of several types of inclusions was also observed and according to the standard ČSN ISO 4967 (420471), the given inclusions exhibited a subcritical size but their distribution close to grain boundaries or directly at grain boundaries can represent the low-energy critical microcrack propagation. Using the method of energy-dispersive spectroscopy, the analysis of the chemical composition from the selected area confirmed that there is mainly the presence of MnS inclusions, especially in the central area of the microstructure (Figure 9). In relation to the microstructure, it was also possible to observe the Al-based oxides and Si-based oxides through the overall longitudinal section area. Moreover, the complex sulfoxides (Figure 10) were visible close to the grain boundaries. Grain boundaries are indicated with arrows.

**Figure 11:**
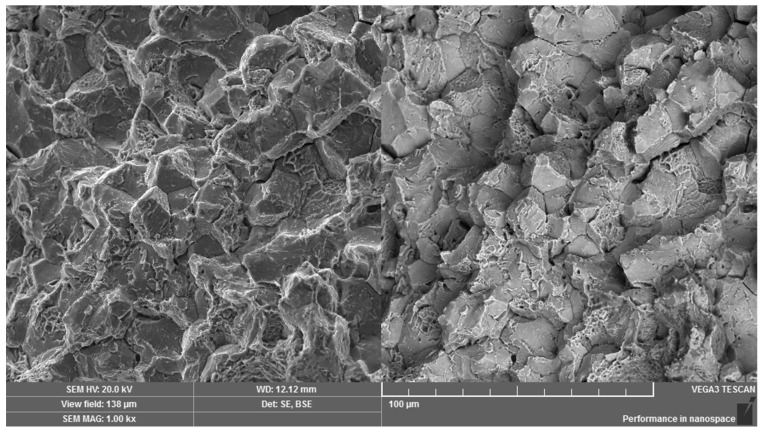
Operational fracture of a high-strength screw under shear loading. Dual representation of the analyzed area: (**left**) SE mode, (**right)** BSE mode.

**Figure 12:**
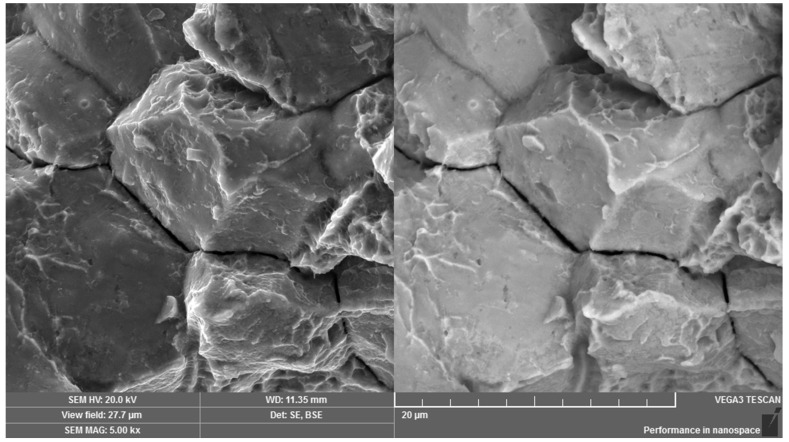
Visible weakness of grain boundaries. Dual representation of the analyzed area: (**left**) SE mode, (**right)** BSE mode.

Scanning electron microscopy in secondary and backscattered electrons mode was used for the fractographic analysis of the fracture surface, relating to a broken screw from practice. Both the peripheral and the central regions were formed mainly by intercrystalline micromechanism with visible pit micromorphology which was observed at revealed grain boundaries (Figure 11). However, the observed pits were shallow with small geometric parameters. The fracture surface exhibited significant decohesion at the grain boundaries (Figure 12) and a large number of intercrystalline microcracks. The fracture surface characteristics correspond to the low-energy propagation of a critical microcrack. In the overall area of the fracture surface, there was no significant direction of the fracture surface due to the shear loading. In addition, there was not any impressed area which could be the consequence of the movement and abrasion of the fracture surfaces.

**Figure 13:**
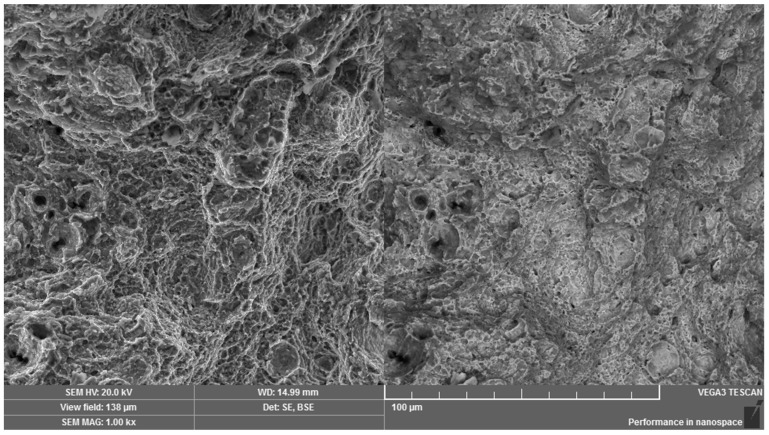
Fracture surface of the reference high-strength screw under tensile loading. Dual representation of the analyzed area: (**left**) SE mode, (**right)** BSE mode.

It is important to point out that the fracture surface of the broken screw did also not exhibit the required degree of toughness and, therefore, further steps of investigation were based on an experimental comparative method, relating to the comparison of the laboratory fracture of the broken screw from practice with the tensile fracture of the identical unbroken screw. The analysis of the fracture surfaces was supplemented by the measurement of the HV1 hardness. Using tensile loading, the fracture surface of the unbroken screw showed a high degree of toughness with the presence of deep pits of the ductile fracture resulting from overloading (Figure 13). The average hardness value from the three measurements for this screw was 372 HV1. The hardness corresponds to the tempering heat treatment of the martensitic structure at a higher temperature and it stands for the microstructure of the upper bainite.

**Figure 14:**
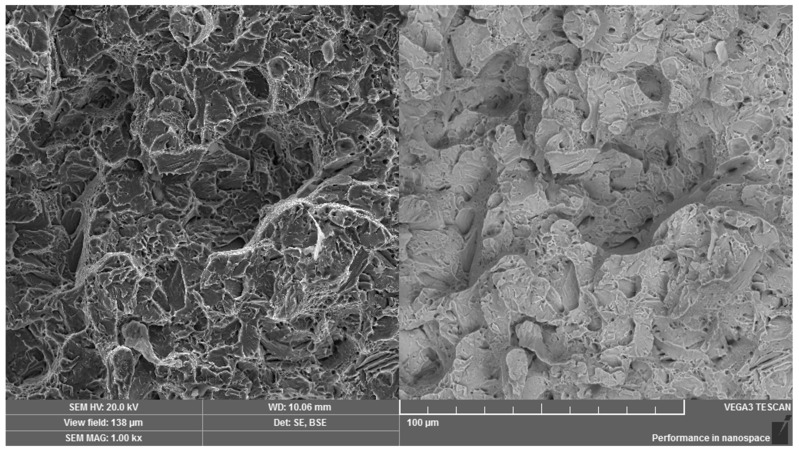
Laboratory fracture of the broken screw in operation after tensile loading. Dual representation of the analyzed area: (**left**) SE mode, (**right)** BSE mode.

**Figure 15:**
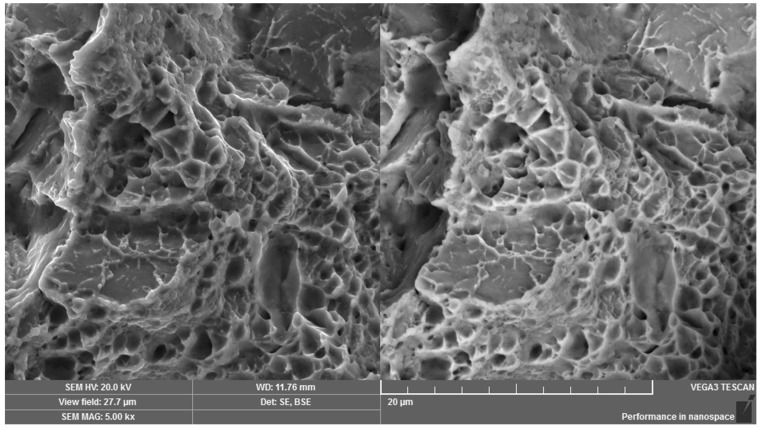
Detail of the pit micromorphology for the broken screw in operation after tensile loading. Dual representation of the analyzed area: (**left**) SE mode, (**right)** BSE mode.

In order to obtain comprehensive information on the fracture properties of the broken screw for the whole volume of its threaded part, a fracture surface under tensile loading was prepared from the rest of the screw in the laboratory. The given fracture surface was mainly formed by a transcrystalline rupture micromechanism (Figure 14) with the visible pit micromorphology. The pits had greater depths and geometric parameters and, moreover, they were represented in greater numbers, which gives important information on high-energy rupture (Figure 15). After tensile loading, the fracture surface showed a higher degree of ductile fracture, but, for a given joining component, it is not an ideal or expected failure in relation to screw overloading and it corresponds to the microstructure of lower bainite with higher hardness values in comparison with the microstructure of the upper bainite. Based on the fractographic analysis and findings, further and additional measurements were performed in relation to HV 1 hardness, which was measured step by step from the edge to the core of the screw [28]. Each area was subjected to three hardness measurements from which the average value was calculated. An average value of 891 HV1 was obtained for the edge, an average value of 796 HV1 was gained for the area between the edge and the center and an average value of 695 HV1 was obtained for the central area of the screw. For evaluation, the Vickers hardness measurement method under HV1 loading used standard ČSN EN ISO 6507-1 Metallic materials—Vickers hardness test—Part 1: Test method. Hardness was measured on the device V10 K/AQ.

## 4. Discussion

Joining or fastening components for highway frame constructions have to meet the strictly given requirements, included in the appropriate standards. The control of individual processing procedures represents a very important role in their production process. If it is not possible to carry out extensive research into the mechanical properties of the defective component, microscopic and fractrographic analysis is widely used. These methods provide valuable information on micropurity, microstructure and fracture properties of materials. The acquired knowledge is directly related to the production and processing of each steel component. On the other hand, the given joining components can be broken in operation and if such a situation occurs the backward analysis of the material processing is required in order to help in the identification of any inappropriate steps or procedures relating to manufacturing process. The investigated broken screw was made of high-strength 41CR4 (1.7035) chrome steel of 10.9 grade with M 27 × 3 dimensions and shank length of 64 mm. Using the method of optical microscopy in combination with the standard series based on standard ČSN ISO 4967 (420471), the analysis of the material micropurity showed that the screw was made of high-purity steel. After etching, oxide inclusions on the basis of Al, Si as well as complex sulfoxides were detected. The given inclusions were subcritical in size but they were distributed close to grain boundaries and it can be dangerous from the point of view of cohesion properties in relation to inappropriate thermal treatment.

According to the technical standard mentioned above, there was the large number of manganese sulfides up to grade 1 in the central area and the given sulfides were distributed in parallel lines. In relation to the observation of the microstructure, the heat treatment of material was based on quenching and a subsequent hardening process. In addition, the hardness value of 695 HV1 was reached in the central area. The micromorphology and hardness correspond to the structure of the lower bainite. Using the methods of scanning electron microscopy and energy-dispersive spectroscopy, the surface Zn layer was identified on the surface of the screw and it represented the anticorrosion protection. On the other side, from the results of the performed investigations, it is important to point out that the given protective layer exhibited a large number of defective areas. According to the thickness of the Zn surface layer, it is possible to assume that it was formed in an electrochemical way by the electroplating process. In the polished state, microcracks were visible in the steel and the propagation of these microcracks was observed from the surface of the threaded part. In addition, the microspace of the microcracks was filled with corrosive products and Zn. In relation to the mentioned microcracks, it is possible to assume that the initiation and subsequent propagation results from the screw production process before the final surface treatment.

The presence of the surface microcracks can have a negative effect on the cohesive strength of the steel metal matrix. From the measured values and the obtained results as well as knowledge, it can be concluded that the main reason for the screw failure was based on the inappropriate heat treatment of steel. In relation to the application of the screw, the required strength of this type of screw can be reached by quenching and tempering up to the upper bainite structure. The mentioned bainitic structure can only be reached by higher tempering temperatures and it results in the required fracture and notch toughness of the material. The chrome steel used to produce the high-strength screw also corresponds to this mentioned process of heat treatment. In addition, the fracture surface of the high-tempered martensite exhibits noticeable pit micromorphology, which stands for high-energy rupture of the material. However, the microstructure of the investigated broken screw was formed by lower bainite and this type of structure represents the increase in hardness values, but it also represents the decrease in toughness values of the material. During the tempering process, the tempering temperature was selected inaccurately and, moreover, the processed material was in the region of low-temperature tempering embrittlement during the given heat treatment. The introduced type of the material embrittlement commonly occurs at temperatures from 350 to 450 °C. Apparently, when a longer tempering time was used, the carbides were dissolved in the bainitic ferrite and subsequently, they were precipitated towards the boundaries of the original austenitic grains. The surface-active elements in the steel can be segregated towards the grain boundaries and it leads to the significant reduction of the cohesive properties in these areas. The occurrence and size of the carbide phase in the bainitic ferrite plates reached higher values and it could cause the increase in the hardness as well as brittleness of this microstructure.

## 5. Conclusions

These facts are also reflected in the character of the fracture surface which was based on low-energy intercrystalline rupture with pit micromorphology in the areas of revealed grain boundaries. An interesting finding is also connected with the results of the comparative method which was based on the comparison of the fracture surfaces of the broken screw under shear operational loading and tensile loading. The given comparative method gives information on the weakness of grain boundaries. The occurrence of weakness was not observed in the whole volume of the screw but it was only in the area of the operational fracture. The rest of the screw volume exhibited the transcrystalline high-energy fracture with the significant pit micromorphology. This character of the fracture surface corresponds to the structure of the lower bainite with higher values of surface hardness. Moreover, the course of HV1 hardness measurements from the edge to the central area indicates an impropriate tempering of screw in overall cross-section because the hardness, which was measured at the edge, was 891 HV1. The given measured hardness value corresponds to the martensitic structure. It can be assumed that there was no optimal loosening over the entire cross-section of the screw. Based on the mentioned facts, it can be concluded that a neither optimum nor effective tempering process was used.

Metallographic and fractographic analysis of broken high-strength screws can contribute to improvement of the measures for the production process and it can lead to the increase in the safety of the surroundings in which the given components are mounted. High-strength screws are used in the assembly of detachable joints of steel structures, which are subject to high strength requirements. These components are used, for example, in construction and steel structures in the transport industry. In the case of a similar fracture of the screw, it would be possible to proceed in the manner as shown in this article. Important information provides knowledge about the microstructure and micromorphology of the fracture surface. It is appropriate if microscopic methods are supplemented by information on the basic mechanical properties of the material. Of course, it is always necessary to look for the cause of the fracture, for whether the structure was not loaded more than the permissible load limit, and for whether the prescribed conditions for tightening the torsions were not violated, etc.

## Figures and Tables

**Table 1:** Chemical composition of high-strength steel (weight %) [26].

Material	C	Si Max.	Mn	P Max.	S Max.	Cr
41Cr4 (1.7035)	0.38–0.45	0.4	0.6–0.9	0.025	0.035	0.9–1.2

**Table 2:** Mechanical properties of high-strength steel [26].

Material	Rm [MPa]	Rp_0.2_ [MPa]	A [%]	HV Min.–Max.	BV Min.–Max.	HR Min.–Max.
41Cr4 (1.7035)	1000	900	9	320–380	304–361	32–39

## Data Availability

No new data were created or analyzed in this study. Data sharing is not applicable to this article.
